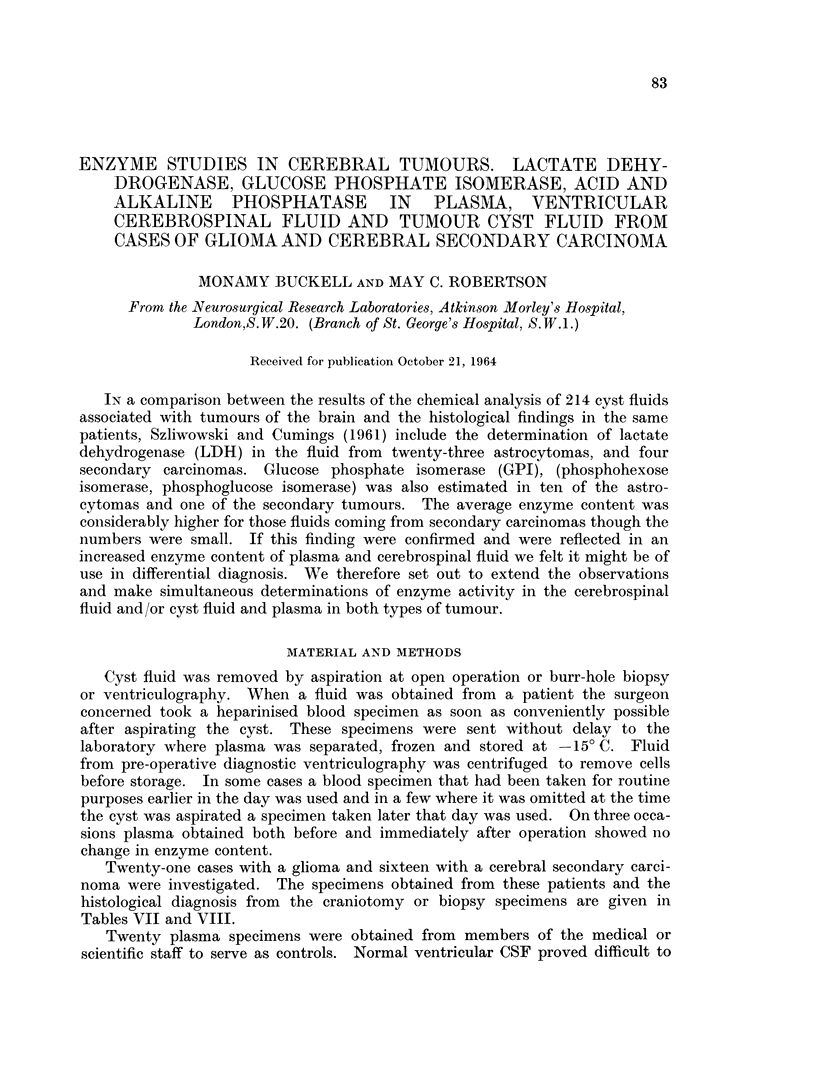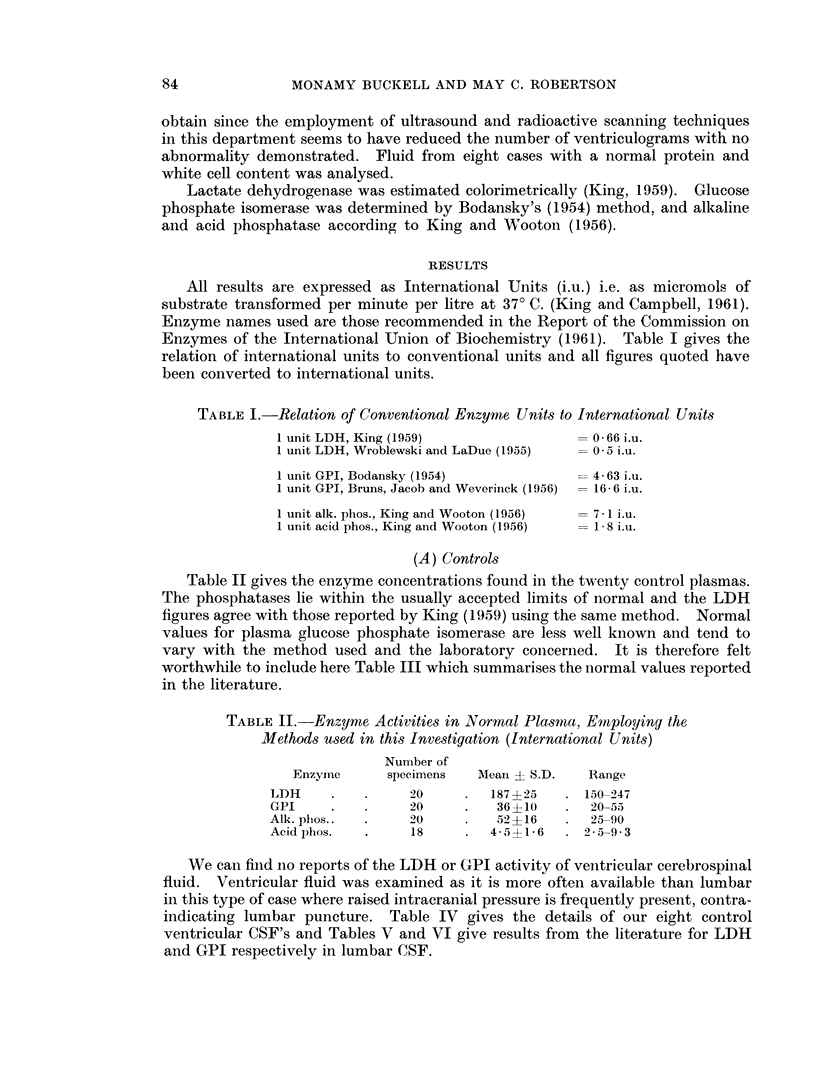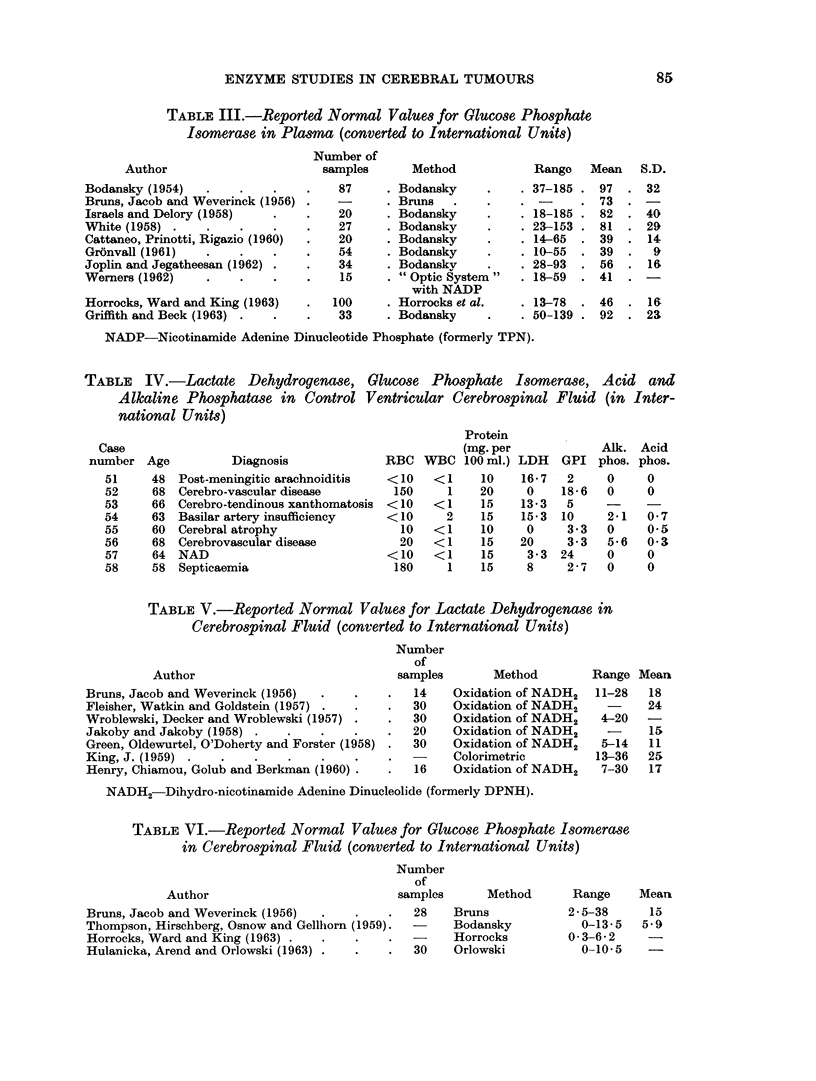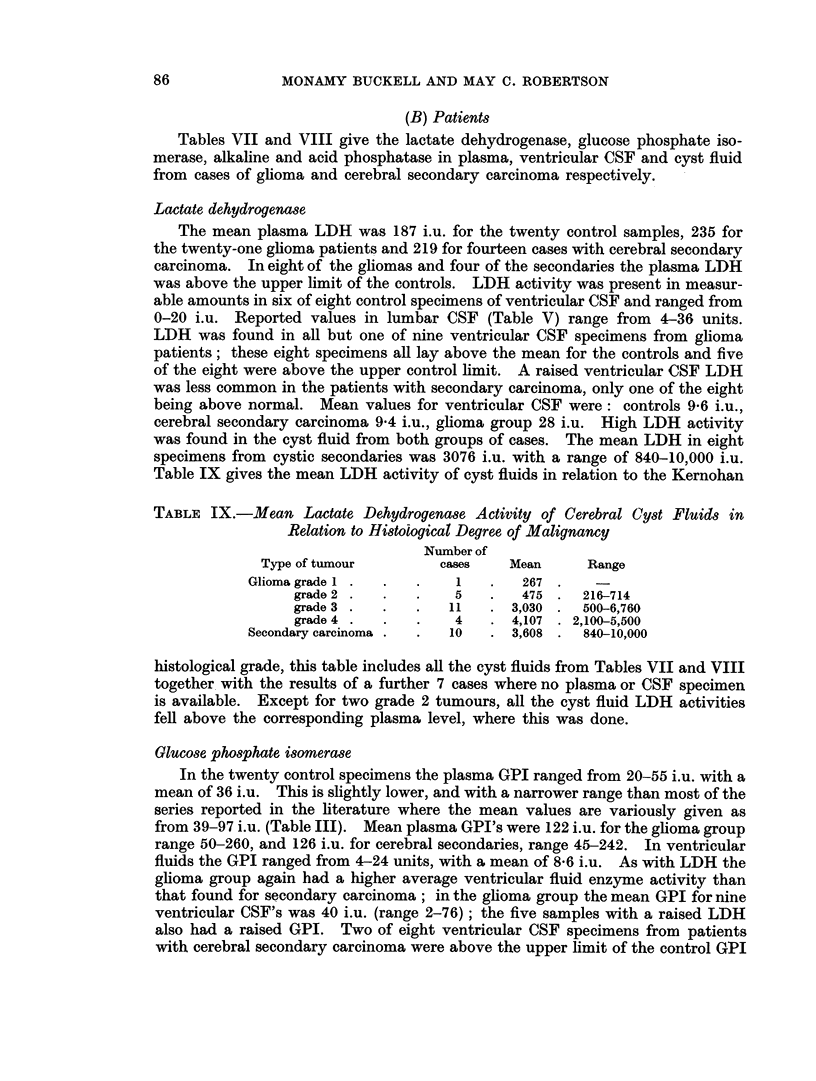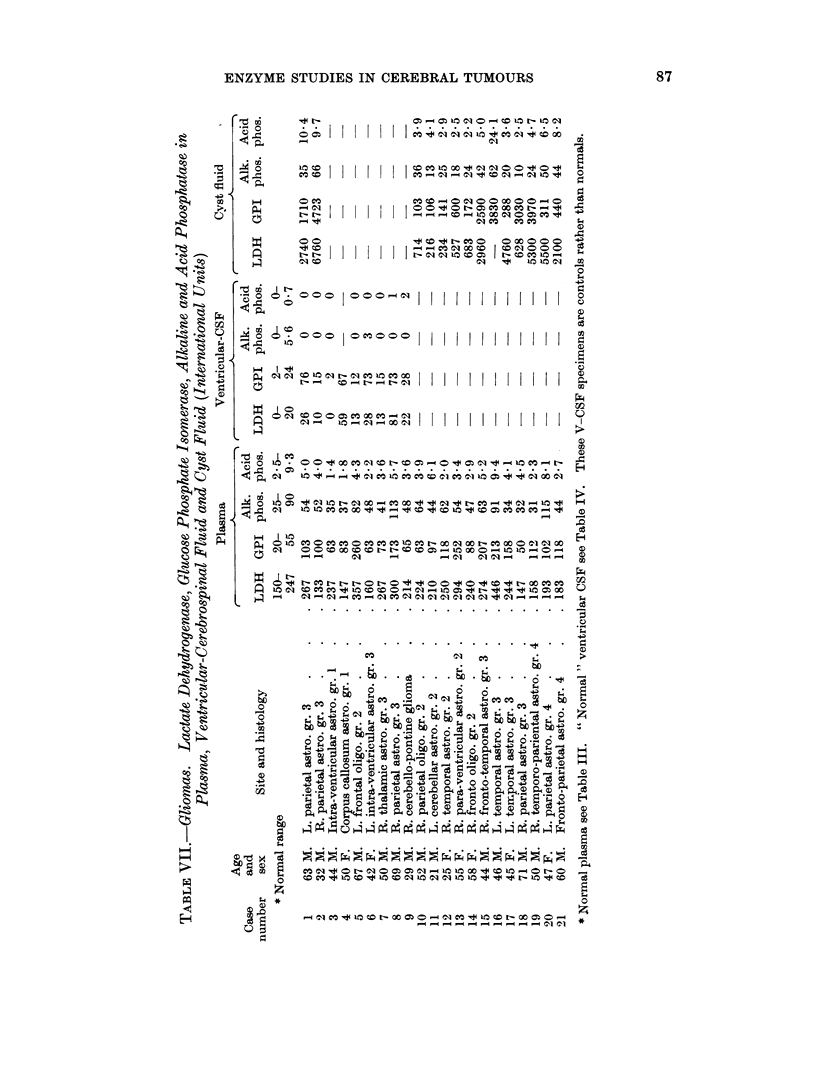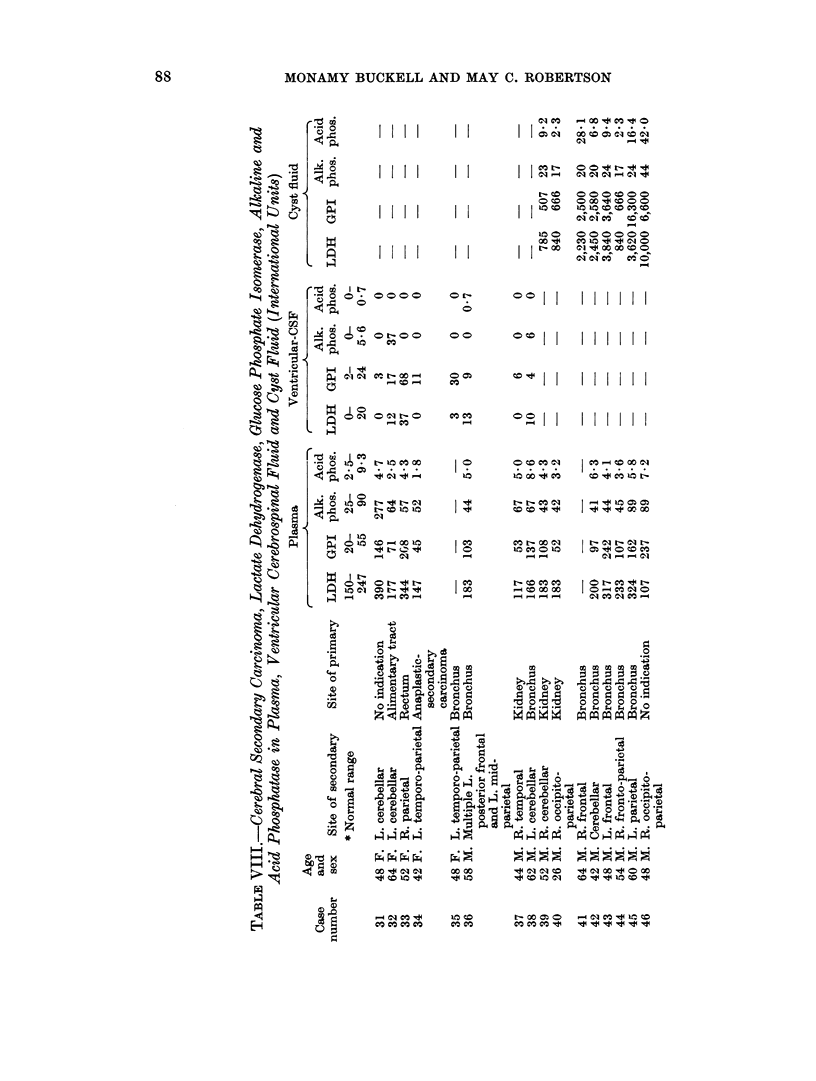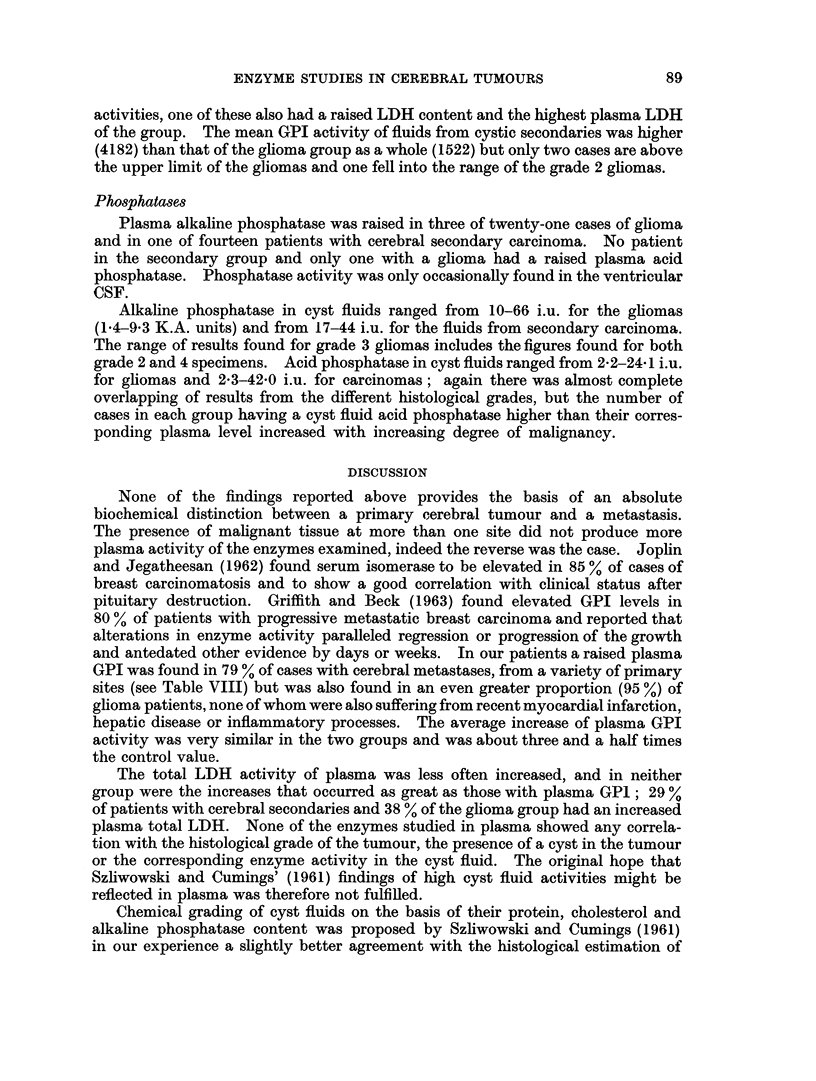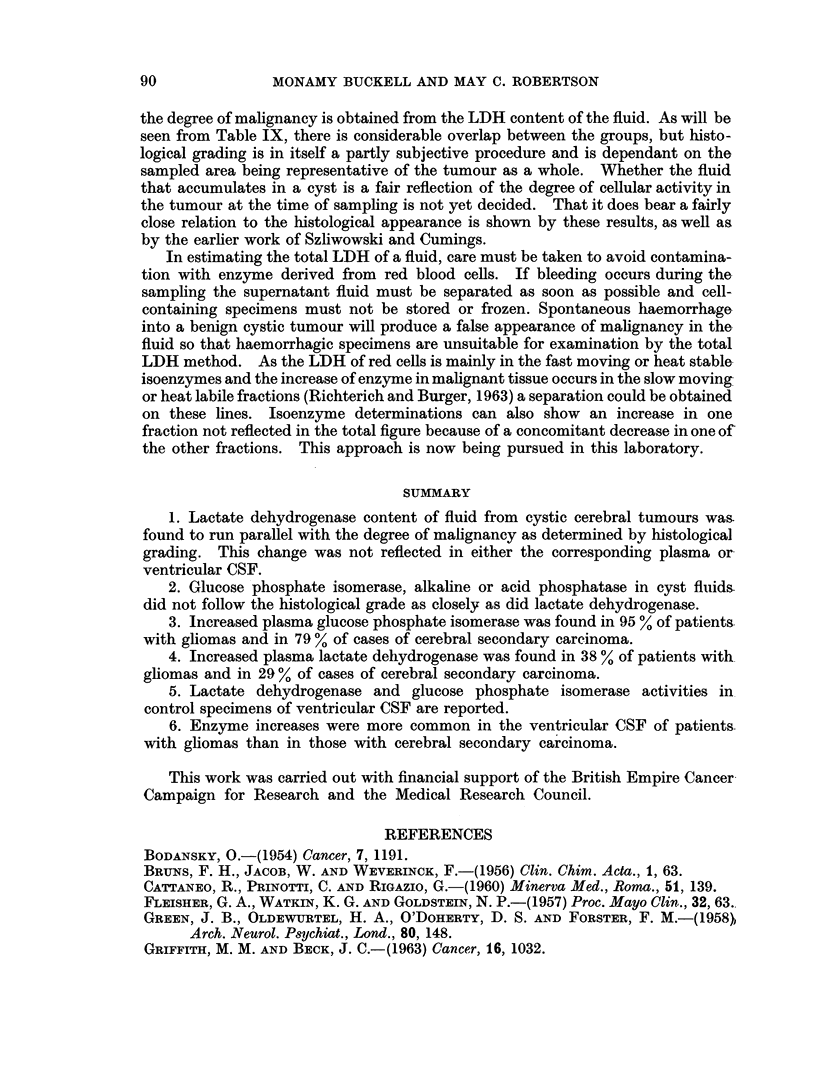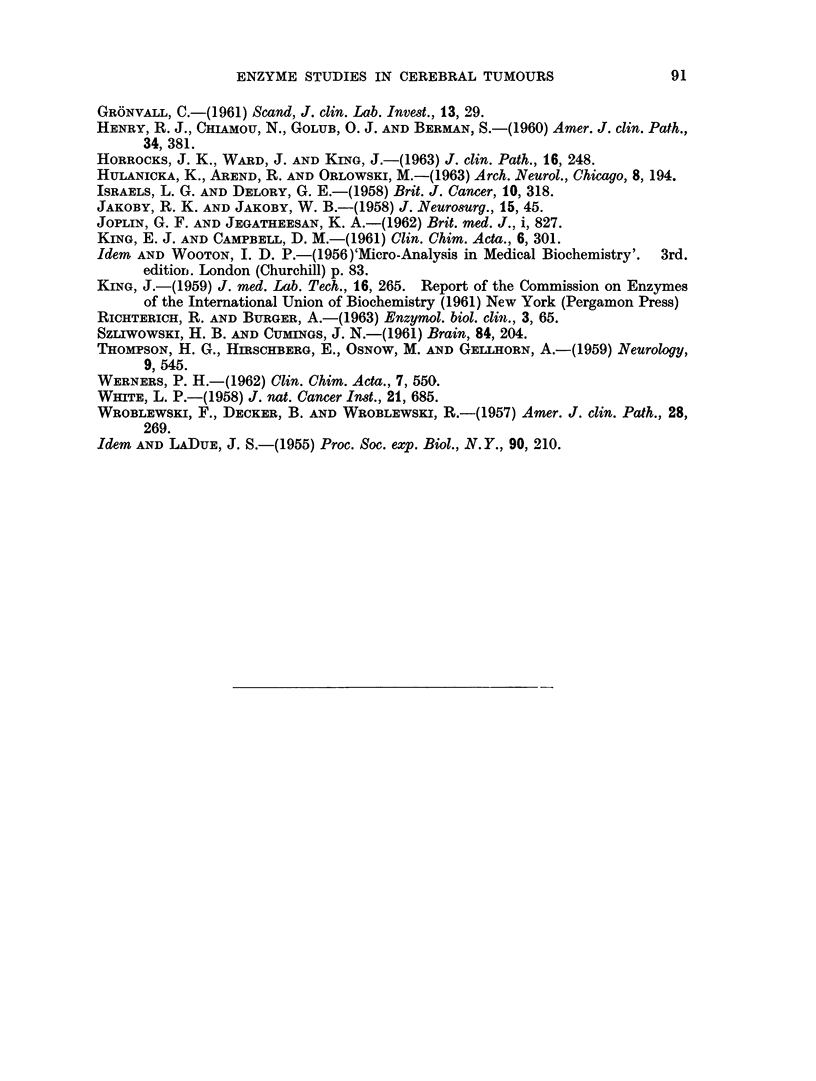# Enzyme Studies in Cerebral Tumours. Lactate Dehydrogenase, Glucose Phosphate Isomerase, Acid and Alkaline Phosphatase in Plasma, Ventricular Cerebrospinal Fluid and Tumour Cyst Fluid from Cases of Glioma and Cerebral Secondary Carcinoma

**DOI:** 10.1038/bjc.1965.8

**Published:** 1965-03

**Authors:** Monamy Buckell, May C. Robertson


### ENZYME STUDIES IN CEREBRAL TUMOURS. LACTATE DEHYDROGENASE, GLUCOSE PHOSPHATE ISOMERASE, ACID AND ALKALINE PHOSPHATASE IN PLASMA, VENTRICULAR CEREBROSPINAL FLUID AND TUMOUR CYST FLUID FROM CASES OF GLIOMA AND CEREBRAL SECONDARY CARCINOMA

MONAMY BUCKELL AND MAY C. ROBERTSON

*From the Neurosurgical Research Laboratories, Atkinson Morley's Hospital, London, S.W.20. (Branch of St. George's Hospital, S.W.1.)*

Received for publication October 21, 1964

In a comparison between the results of the chemical analysis of 214 cyst fluids associated with tumours of the brain and the histological findings in the same patients, Szliwowski and Cumings (1961) include the determination of lactate dehydrogenase (LDH) in the fluid from twenty-three astrocytomas, and four secondary carcinomas. Glucose phosphate isomerase (GPI), (phosphohexose isomerase, phosphoglucose isomerase) was also estimated in ten of the astrocytomas and one of the secondary tumours. The average enzyme content was considerably higher for those fluids coming from secondary carcinomas though the numbers were small. If this finding were confirmed and were reflected in an increased enzyme content of plasma and cerebrospinal fluid we felt it might be of use in differential diagnosis. We therefore set out to extend the observations and make simultaneous determinations of enzyme activity in the cerebrospinal fluid and/or cyst fluid and plasma in both types of tumour.

### MATERIAL AND METHODS

Cyst fluid was removed by aspiration at open operation or burr-hole biopsy or ventriculography. When a fluid was obtained from a patient the surgeon concerned took a heparinised blood specimen as soon as conveniently possible after aspirating the cyst. These specimens were sent without delay to the laboratory where plasma was separated, frozen and stored at $-15^{\circ}$ C. Fluid from pre-operative diagnostic ventriculography was centrifuged to remove cells before storage. In some cases a blood specimen that had been taken for routine purposes earlier in the day was used and in a few where it was omitted at the time the cyst was aspirated a specimen taken later that day was used. On three occasions plasma obtained both before and immediately after operation showed no change in enzyme content.

Twenty-one cases with a glioma and sixteen with a cerebral secondary carcinoma were investigated. The specimens obtained from these patients and the histological diagnosis from the craniotomy or biopsy specimens are given in Tables VII and VIII.

Twenty plasma specimens were obtained from members of the medical or scientific staff to serve as controls. Normal ventricular CSF proved difficult to obtain since the employment of ultrasound and radioactive scanning techniques in this department seems to have reduced the number of ventriculograms with no abnormality demonstrated. Fluid from eight cases with a normal protein and white cell content was analysed.

Lactate dehydrogenase was estimated colorimetrically (King, 1959). Glucose phosphate isomerase was determined by Bodansky's (1954) method, and alkaline and acid phosphatase according to King and Wooton (1956).

### RESULTS

All results are expressed as International Units (i.u.) i.e. as micromols of substrate transformed per minute per litre at 37° C. (King and Campbell, 1961). Enzyme names used are those recommended in the Report of the Commission on Enzymes of the International Union of Biochemistry (1961). Table I gives the relation of international units to conventional units and all figures quoted have been converted to international units.

TABLE I.—*Relation of Conventional Enzyme Units to International Units*

| | |
|---|---|
| 1 unit LDH, King (1959) | = 0·66 i.u. |
| 1 unit LDH, Wroblewski and LaDue (1955) | = 0·5 i.u. |
| 1 unit GPI, Bodansky (1954) | = 4·63 i.u. |
| 1 unit GPI, Bruns, Jacob and Weverinck (1956) | = 16·6 i.u. |
| 1 unit alk. phos., King and Wooton (1956) | = 7·1 i.u. |
| 1 unit acid phos., King and Wooton (1956) | = 1·8 i.u. |

### (*A*) *Controls*

Table II gives the enzyme concentrations found in the twenty control plasmas. The phosphatases lie within the usually accepted limits of normal and the LDH figures agree with those reported by King (1959) using the same method. Normal values for plasma glucose phosphate isomerase are less well known and tend to vary with the method used and the laboratory concerned. It is therefore felt worthwhile to include here Table III which summarises the normal values reported in the literature.

TABLE II.—*Enzyme Activities in Normal Plasma, Employing the Methods used in this Investigation (International Units)*

| Enzyme | Number of specimens | Mean ± S.D. | Range |
|---|---|---|---|
| LDH | 20 | 187±25 | 150–247 |
| GPI | 20 | 36±10 | 20–55 |
| Alk. phos. | 20 | 52±16 | 25–90 |
| Acid phos. | 18 | 4·5±1·6 | 2·5–9·3 |

We can find no reports of the LDH or GPI activity of ventricular cerebrospinal fluid. Ventricular fluid was examined as it is more often available than lumbar in this type of case where raised intracranial pressure is frequently present, contraindicating lumbar puncture. Table IV gives the details of our eight control ventricular CSF's and Tables V and VI give results from the literature for LDH and GPI respectively in lumbar CSF.

TABLE III.—*Reported Normal Values for Glucose Phosphate Isomerase in Plasma (converted to International Units)*

| Author | Number of samples | Method | Range | Mean | S.D. |
|---|---|---|---|---|---|
| Bodansky (1954) | 87 | Bodansky | 37–185 | 97 | 32 |
| Bruns, Jacob and Weverinck (1956) | — | Bruns | — | 73 | — |
| Israels and Delory (1958) | 20 | Bodansky | 18–185 | 82 | 40 |
| White (1958) | 27 | Bodansky | 23–153 | 81 | 29 |
| Cattaneo, Prinotti, Rigazio (1960) | 20 | Bodansky | 14–65 | 39 | 14 |
| Grönvall (1961) | 54 | Bodansky | 10–55 | 39 | 9 |
| Joplin and Jegatheesan (1962) | 34 | Bodansky | 28–93 | 56 | 16 |
| Werners (1962) | 15 | " Optic System " with NADP | 18–59 | 41 | — |
| Horrocks, Ward and King (1963) | 100 | Horrocks *et al.* | 13–78 | 46 | 16 |
| Griffith and Beck (1963) | 33 | Bodansky | 50–139 | 92 | 23 |

NADP—Nicotinamide Adenine Dinucleotide Phosphate (formerly TPN).

TABLE IV.—*Lactate Dehydrogenase, Glucose Phosphate Isomerase, Acid and Alkaline Phosphatase in Control Ventricular Cerebrospinal Fluid (in International Units)*

| Case number | Age | Diagnosis | RBC | WBC | Protein (mg. per 100 ml.) | LDH | GPI | Alk. phos. | Acid phos. |
|---|---|---|---|---|---|---|---|---|---|
| 51 | 48 | Post-meningitic arachnoiditis | <10 | <1 | 10 | 16·7 | 2 | 0 | 0 |
| 52 | 68 | Cerebro-vascular disease | 150 | 1 | 20 | 0 | 18·6 | 0 | 0 |
| 53 | 66 | Cerebro-tendinous xanthomatosis | <10 | <1 | 15 | 13·3 | 5 | — | — |
| 54 | 63 | Basilar artery insufficiency | <10 | 2 | 15 | 15·3 | 10 | 2·1 | 0·7 |
| 55 | 60 | Cerebral atrophy | 10 | <1 | 10 | 0 | 3·3 | 0 | 0·5 |
| 56 | 68 | Cerebrovascular disease | 20 | <1 | 15 | 20 | 3·3 | 5·6 | 0·3 |
| 57 | 64 | NAD | <10 | <1 | 15 | 3·3 | 24 | 0 | 0 |
| 58 | 58 | Septicaemia | 180 | 1 | 15 | 8 | 2·7 | 0 | 0 |

TABLE V.—*Reported Normal Values for Lactate Dehydrogenase in Cerebrospinal Fluid (converted to International Units)*

| Author | Number of samples | Method | Range | Mean |
|---|---|---|---|---|
| Bruns, Jacob and Weverinck (1956) | 14 | Oxidation of $NADH_2$ | 11–28 | 18 |
| Fleisher, Watkin and Goldstein (1957) | 30 | Oxidation of $NADH_2$ | — | 24 |
| Wroblewski, Decker and Wroblewski (1957) | 30 | Oxidation of $NADH_2$ | 4–20 | — |
| Jakoby and Jakoby (1958) | 20 | Oxidation of $NADH_2$ | — | 15 |
| Green, Oldewurtel, O'Doherty and Forster (1958) | 30 | Oxidation of $NADH_2$ | 5–14 | 11 |
| King, J. (1959) | — | Colorimetric | 13–36 | 25 |
| Henry, Chiamou, Golub and Berkman (1960) | 16 | Oxidation of $NADH_2$ | 7–30 | 17 |

$NADH_2$—Dihydro-nicotinamide Adenine Dinucleolide (formerly DPNH).

TABLE VI.—*Reported Normal Values for Glucose Phosphate Isomerase in Cerebrospinal Fluid (converted to International Units)*

| Author | Number of samples | Method | Range | Mean |
|---|---|---|---|---|
| Bruns, Jacob and Weverinck (1956) | 28 | Bruns | 2·5–38 | 15 |
| Thompson, Hirschberg, Osnow and Gellhorn (1959) | — | Bodansky | 0–13·5 | 5·9 |
| Horrocks, Ward and King (1963) | — | Horrocks | 0·3–6·2 | — |
| Hulanicka, Arend and Orlowski (1963) | 30 | Orlowski | 0–10·5 | — |

### (*B*) *Patients*

Tables VII and VIII give the lactate dehydrogenase, glucose phosphate isomerase, alkaline and acid phosphatase in plasma, ventricular CSF and cyst fluid from cases of glioma and cerebral secondary carcinoma respectively.

### *Lactate dehydrogenase*

The mean plasma LDH was 187 i.u. for the twenty control samples, 235 for the twenty-one glioma patients and 219 for fourteen cases with cerebral secondary carcinoma. In eight of the gliomas and four of the secondaries the plasma LDH was above the upper limit of the controls. LDH activity was present in measurable amounts in six of eight control specimens of ventricular CSF and ranged from 0–20 i.u. Reported values in lumbar CSF (Table V) range from 4–36 units. LDH was found in all but one of nine ventricular CSF specimens from glioma patients; these eight specimens all lay above the mean for the controls and five of the eight were above the upper control limit. A raised ventricular CSF LDH was less common in the patients with secondary carcinoma, only one of the eight being above normal. Mean values for ventricular CSF were: controls 9·6 i.u., cerebral secondary carcinoma 9·4 i.u., glioma group 28 i.u. High LDH activity was found in the cyst fluid from both groups of cases. The mean LDH in eight specimens from cystic secondaries was 3076 i.u. with a range of 840–10,000 i.u. Table IX gives the mean LDH activity of cyst fluids in relation to the Kernohan

TABLE IX.—*Mean Lactate Dehydrogenase Activity of Cerebral Cyst Fluids in Relation to Histological Degree of Malignancy*

| Type of tumour | Number of cases | Mean | Range |
|---|---|---|---|
| Glioma grade 1 | 1 | 267 | — |
| grade 2 | 5 | 475 | 216–714 |
| grade 3 | 11 | 3,030 | 500–6,760 |
| grade 4 | 4 | 4,107 | 2,100–5,500 |
| Secondary carcinoma | 10 | 3,608 | 840–10,000 |

histological grade, this table includes all the cyst fluids from Tables VII and VIII together with the results of a further 7 cases where no plasma or CSF specimen is available. Except for two grade 2 tumours, all the cyst fluid LDH activities fell above the corresponding plasma level, where this was done.

### *Glucose phosphate isomerase*

In the twenty control specimens the plasma GPI ranged from 20–55 i.u. with a mean of 36 i.u. This is slightly lower, and with a narrower range than most of the series reported in the literature where the mean values are variously given as from 39–97 i.u. (Table III). Mean plasma GPI's were 122 i.u. for the glioma group range 50–260, and 126 i.u. for cerebral secondaries, range 45–242. In ventricular fluids the GPI ranged from 4–24 units, with a mean of 8·6 i.u. As with LDH the glioma group again had a higher average ventricular fluid enzyme activity than that found for secondary carcinoma; in the glioma group the mean GPI for nine ventricular CSF's was 40 i.u. (range 2–76); the five samples with a raised LDH also had a raised GPI. Two of eight ventricular CSF specimens from patients with cerebral secondary carcinoma were above the upper limit of the control GPI

TABLE VII.—*Gliomas. Lactate Dehydrogenase, Glucose Phosphate Isomerase, Alkaline and Acid Phosphatase in Plasma, Ventricular-Cerebrospinal Fluid and Cyst Fluid (International Units)*

| Case number | Age and sex | Site and histology | Plasma LDH | Plasma GPI | Plasma Alk. phos. | Plasma Acid phos. | Ventricular-CSF LDH | Ventricular-CSF GPI | Ventricular-CSF Alk. phos. | Ventricular-CSF Acid phos. | Cyst fluid LDH | Cyst fluid GPI | Cyst fluid Alk. phos. | Cyst fluid Acid phos. |
|---|---|---|---|---|---|---|---|---|---|---|---|---|---|---|
| | * Normal range | | 150–247 | 20–55 | 25–90 | 2·5–9·3 | 0–20 | 2–24 | 0–5·6 | 0–0·7 | | | | |
| 1 | 63 M. | L. parietal astro. gr. 3 | 267 | 103 | 54 | 5·0 | 26 | 76 | 0 | 0 | 2740 | 1710 | 35 | 10·4 |
| 2 | 32 M. | R. parietal astro. gr. 3 | 133 | 100 | 52 | 4·0 | 10 | 15 | 0 | 0 | 6760 | 4723 | 66 | 9·7 |
| 3 | 44 M. | Intra-ventricular astro. gr. 1 | 237 | 63 | 35 | 1·4 | 0 | 2 | 0 | 0 | — | — | — | — |
| 4 | 50 F. | Corpus callosum astro. gr. 1 | 147 | 83 | 37 | 1·8 | 59 | 67 | — | — | — | — | — | — |
| 5 | 67 M. | L. frontal oligo. gr. 2 | 357 | 260 | 82 | 4·3 | 13 | 12 | 0 | 0 | — | — | — | — |
| 6 | 42 F. | L. intra-ventricular astro. gr. 3 | 160 | 63 | 48 | 2·2 | 28 | 73 | 3 | 0 | — | — | — | — |
| 7 | 50 M. | R. thalamic astro. gr. 3 | 267 | 73 | 41 | 3·6 | 13 | 15 | 0 | 0 | — | — | — | — |
| 8 | 69 M. | R. parietal astro. gr. 3 | 300 | 173 | 113 | 5·7 | 81 | 73 | 0 | 1 | — | — | — | — |
| 9 | 29 M. | R. cerebello-pontine glioma | 214 | 65 | 48 | 3·6 | 22 | 28 | 0 | 2 | — | — | — | — |
| 10 | 52 M. | R. parietal oligo. gr. 2 | 224 | 63 | 64 | 3·9 | — | — | — | — | 714 | 103 | 36 | 3·9 |
| 11 | 21 M. | L. cerebellar astro. gr. 2 | 210 | 97 | 44 | 6·1 | — | — | — | — | 216 | 106 | 13 | 4·1 |
| 12 | 25 F. | R. temporal astro. gr. 2 | 250 | 118 | 62 | 2·0 | — | — | — | — | 234 | 141 | 25 | 2·9 |
| 13 | 55 F. | R. para-ventricular astro. gr. 2 | 294 | 252 | 54 | 3·4 | — | — | — | — | 527 | 600 | 18 | 2·5 |
| 14 | 58 F. | R. fronto oligo. gr. 2 | 240 | 88 | 47 | 2·9 | — | — | — | — | 683 | 172 | 24 | 2·2 |
| 15 | 44 M. | R. fronto-temporal astro. gr. 3 | 274 | 207 | 63 | 5·2 | — | — | — | — | 2960 | 2590 | 42 | 5·0 |
| 16 | 46 M. | L. temporal astro. gr. 3 | 446 | 213 | 91 | 9·4 | — | — | — | — | — | 3830 | 62 | 24·1 |
| 17 | 45 F. | L. temporal astro. gr. 3 | 244 | 158 | 34 | 4·1 | — | — | — | — | 4760 | 288 | 20 | 3·6 |
| 18 | 71 M. | R. parietal astro. gr. 3 | 147 | 50 | 32 | 4·5 | — | — | — | — | 628 | 3030 | 10 | 2·5 |
| 19 | 50 M. | R. temporo-pariental astro. gr. 4 | 158 | 112 | 31 | 2·3 | — | — | — | — | 5300 | 3970 | 24 | 4·7 |
| 20 | 47 F. | L. parietal astro. gr. 4 | 193 | 102 | 115 | 8·1 | — | — | — | — | 5500 | 311 | 50 | 6·5 |
| 21 | 60 M. | Fronto-parietal astro. gr. 4 | 183 | 118 | 44 | 2·7 | — | — | — | — | 2100 | 440 | 44 | 8·2 |

* Normal plasma see Table III. " Normal " ventricular CSF see Table IV. These V–CSF specimens are controls rather than normals.

TABLE VIII.—*Cerebral Secondary Carcinoma, Lactate Dehydrogenase, Glucose Phosphate Isomerase, Alkaline and Acid Phosphatase in Plasma, Ventricular Cerebrospinal Fluid and Cyst Fluid (International Units)*

| | | | | Plasma | | | | Ventricular-CSF | | | | Cyst fluid | | | |
|---|---|---|---|---|---|---|---|---|---|---|---|---|---|---|---|
| Case number | Age and sex | Site of secondary | Site of primary | LDH | GPI | Alk. phos. | Acid phos. | LDH | GPI | Alk. phos. | Acid phos. | LDH | GPI | Alk. phos. | Acid phos. |
| | | * Normal range | | 150–247 | 20–55 | 25–90 | 2·5–9·3 | 0–20 | 2–24 | 0–5·6 | 0–0·7 | | | | |
| 31 | 48 F. | L. cerebellar | No indication | 390 | 146 | 277 | 4·7 | 0 | 3 | 0 | 0 | — | — | — | — |
| 32 | 64 F. | L. cerebellar | Alimentary tract | 177 | 71 | 64 | 2·5 | 12 | 17 | 37 | 0 | — | — | — | — |
| 33 | 52 F. | R. parietal | Rectum | 344 | 208 | 57 | 4·3 | 37 | 68 | 0 | 0 | — | — | — | — |
| 34 | 42 F. | L. temporo-parietal | Anaplastic-secondary carcinoma | 147 | 45 | 52 | 1·8 | 0 | 11 | 0 | 0 | — | — | — | — |
| 35 | 48 F. | L. temporo-parietal | Bronchus | — | — | — | — | 3 | 30 | 0 | 0 | — | — | — | — |
| 36 | 58 M. | Multiple L. posterior frontal and L. mid-parietal | Bronchus | 183 | 103 | 44 | 5·0 | 13 | 9 | 0 | 0·7 | — | — | — | — |
| 37 | 44 M. | R. temporal | Kidney | 117 | 53 | 67 | 5·0 | 0 | 6 | 0 | 0 | — | — | — | — |
| 38 | 62 M. | L. cerebellar | Bronchus | 166 | 137 | 67 | 8·6 | 10 | 4 | 6 | 0 | — | — | — | — |
| 39 | 52 M. | R. cerebellar | Kidney | 183 | 108 | 43 | 4·3 | — | — | — | — | 785 | 507 | 23 | 9·2 |
| 40 | 26 M. | R. occipito-parietal | Kidney | 183 | 52 | 42 | 3·2 | — | — | — | — | 840 | 666 | 17 | 2·3 |
| 41 | 64 M. | R. frontal | Bronchus | — | — | — | — | — | — | — | — | 2,230 | 2,500 | 20 | 28·1 |
| 42 | 42 M. | Cerebellar | Bronchus | 200 | 97 | 41 | 6·3 | — | — | — | — | 2,450 | 2,580 | 20 | 6·8 |
| 43 | 48 M. | L. frontal | Bronchus | 317 | 242 | 44 | 4·1 | — | — | — | — | 3,840 | 3,640 | 24 | 9·4 |
| 44 | 54 M. | R. fronto-parietal | Bronchus | 233 | 107 | 45 | 3·6 | — | — | — | — | 840 | 666 | 17 | 2·3 |
| 45 | 60 M. | L. parietal | Bronchus | 324 | 162 | 89 | 5·8 | — | — | — | — | 3,620 | 16,300 | 24 | 16·4 |
| 46 | 48 M. | R. occipito-parietal | No indication | 107 | 237 | 89 | 7·2 | — | — | — | — | 10,000 | 6,600 | 44 | 42·0 |

activities, one of these also had a raised LDH content and the highest plasma LDH of the group. The mean GPI activity of fluids from cystic secondaries was higher (4182) than that of the glioma group as a whole (1522) but only two cases are above the upper limit of the gliomas and one fell into the range of the grade 2 gliomas.

*Phosphatases*

Plasma alkaline phosphatase was raised in three of twenty-one cases of glioma and in one of fourteen patients with cerebral secondary carcinoma. No patient in the secondary group and only one with a glioma had a raised plasma acid phosphatase. Phosphatase activity was only occasionally found in the ventricular CSF.

Alkaline phosphatase in cyst fluids ranged from 10–66 i.u. for the gliomas (1·4–9·3 K.A. units) and from 17–44 i.u. for the fluids from secondary carcinoma. The range of results found for grade 3 gliomas includes the figures found for both grade 2 and 4 specimens. Acid phosphatase in cyst fluids ranged from 2·2–24·1 i.u. for gliomas and 2·3–42·0 i.u. for carcinomas ; again there was almost complete overlapping of results from the different histological grades, but the number of cases in each group having a cyst fluid acid phosphatase higher than their corresponding plasma level increased with increasing degree of malignancy.

### DISCUSSION

None of the findings reported above provides the basis of an absolute biochemical distinction between a primary cerebral tumour and a metastasis. The presence of malignant tissue at more than one site did not produce more plasma activity of the enzymes examined, indeed the reverse was the case. Joplin and Jegatheesan (1962) found serum isomerase to be elevated in 85 % of cases of breast carcinomatosis and to show a good correlation with clinical status after pituitary destruction. Griffith and Beck (1963) found elevated GPI levels in 80 % of patients with progressive metastatic breast carcinoma and reported that alterations in enzyme activity paralleled regression or progression of the growth and antedated other evidence by days or weeks. In our patients a raised plasma GPI was found in 79 % of cases with cerebral metastases, from a variety of primary sites (see Table VIII) but was also found in an even greater proportion (95 %) of glioma patients, none of whom were also suffering from recent myocardial infarction, hepatic disease or inflammatory processes. The average increase of plasma GPI activity was very similar in the two groups and was about three and a half times the control value.

The total LDH activity of plasma was less often increased, and in neither group were the increases that occurred as great as those with plasma GPI ; 29 % of patients with cerebral secondaries and 38 % of the glioma group had an increased plasma total LDH. None of the enzymes studied in plasma showed any correlation with the histological grade of the tumour, the presence of a cyst in the tumour or the corresponding enzyme activity in the cyst fluid. The original hope that Szliwowski and Cumings' (1961) findings of high cyst fluid activities might be reflected in plasma was therefore not fulfilled.

Chemical grading of cyst fluids on the basis of their protein, cholesterol and alkaline phosphatase content was proposed by Szliwowski and Cumings (1961) in our experience a slightly better agreement with the histological estimation of the degree of malignancy is obtained from the LDH content of the fluid. As will be seen from Table IX, there is considerable overlap between the groups, but histological grading is in itself a partly subjective procedure and is dependant on the sampled area being representative of the tumour as a whole. Whether the fluid that accumulates in a cyst is a fair reflection of the degree of cellular activity in the tumour at the time of sampling is not yet decided. That it does bear a fairly close relation to the histological appearance is shown by these results, as well as by the earlier work of Szliwowski and Cumings.

In estimating the total LDH of a fluid, care must be taken to avoid contamination with enzyme derived from red blood cells. If bleeding occurs during the sampling the supernatant fluid must be separated as soon as possible and cell-containing specimens must not be stored or frozen. Spontaneous haemorrhage into a benign cystic tumour will produce a false appearance of malignancy in the fluid so that haemorrhagic specimens are unsuitable for examination by the total LDH method. As the LDH of red cells is mainly in the fast moving or heat stable isoenzymes and the increase of enzyme in malignant tissue occurs in the slow moving or heat labile fractions (Richterich and Burger, 1963) a separation could be obtained on these lines. Isoenzyme determinations can also show an increase in one fraction not reflected in the total figure because of a concomitant decrease in one of the other fractions. This approach is now being pursued in this laboratory.

### SUMMARY

1. Lactate dehydrogenase content of fluid from cystic cerebral tumours was found to run parallel with the degree of malignancy as determined by histological grading. This change was not reflected in either the corresponding plasma or ventricular CSF.

2. Glucose phosphate isomerase, alkaline or acid phosphatase in cyst fluids did not follow the histological grade as closely as did lactate dehydrogenase.

3. Increased plasma glucose phosphate isomerase was found in 95 % of patients with gliomas and in 79 % of cases of cerebral secondary carcinoma.

4. Increased plasma lactate dehydrogenase was found in 38 % of patients with gliomas and in 29 % of cases of cerebral secondary carcinoma.

5. Lactate dehydrogenase and glucose phosphate isomerase activities in control specimens of ventricular CSF are reported.

6. Enzyme increases were more common in the ventricular CSF of patients with gliomas than in those with cerebral secondary carcinoma.

This work was carried out with financial support of the British Empire Cancer Campaign for Research and the Medical Research Council.

### REFERENCES

BODANSKY, O.—(1954) *Cancer*, **7**, 1191.

BRUNS, F. H., JACOB, W. AND WEVERINCK, F.—(1956) *Clin. Chim. Acta.*, **1**, 63.

CATTANEO, R., PRINOTTI, C. AND RIGAZIO, G.—(1960) *Minerva Med., Roma.*, **51**, 139.

FLEISHER, G. A., WATKIN, K. G. AND GOLDSTEIN, N. P.—(1957) *Proc. Mayo Clin.*, **32**, 63.

GREEN, J. B., OLDEWURTEL, H. A., O'DOHERTY, D. S. AND FORSTER, F. M.—(1958) *Arch. Neurol. Psychiat., Lond.*, **80**, 148.

GRIFFITH, M. M. AND BECK, J. C.—(1963) *Cancer*, **16**, 1032.

Grönvall, C.—(1961) *Scand, J. clin. Lab. Invest.*, **13**, 29.
Henry, R. J., Chiamou, N., Golub, O. J. and Berman, S.—(1960) *Amer. J. clin. Path.*, **34**, 381.
Horrocks, J. K., Ward, J. and King, J.—(1963) *J. clin. Path.*, **16**, 248.
Hulanicka, K., Arend, R. and Orlowski, M.—(1963) *Arch. Neurol., Chicago*, **8**, 194.
Israels, L. G. and Delory, G. E.—(1958) *Brit. J. Cancer*, **10**, 318.
Jakoby, R. K. and Jakoby, W. B.—(1958) *J. Neurosurg.*, **15**, 45.
Joplin, G. F. and Jegatheesan, K. A.—(1962) *Brit. med. J.*, i, 827.
King, E. J. and Campbell, D. M.—(1961) *Clin. Chim. Acta.*, **6**, 301.
*Idem* and Wooton, I. D. P.—(1956)'Micro-Analysis in Medical Biochemistry'. 3rd. edition. London (Churchill) p. 83.
King, J.—(1959) *J. med. Lab. Tech.*, **16**, 265. Report of the Commission on Enzymes of the International Union of Biochemistry (1961) New York (Pergamon Press)
Richterich, R. and Burger, A.—(1963) *Enzymol. biol. clin.*, **3**, 65.
Szliwowski, H. B. and Cumings, J. N.—(1961) *Brain*, **84**, 204.
Thompson, H. G., Hirschberg, E., Osnow, M. and Gellhorn, A.—(1959) *Neurology*, **9**, 545.
Werners, P. H.—(1962) *Clin. Chim. Acta.*, **7**, 550.
White, L. P.—(1958) *J. nat. Cancer Inst.*, **21**, 685.
Wroblewski, F., Decker, B. and Wroblewski, R.—(1957) *Amer. J. clin. Path.*, **28**, 269.
*Idem* and LaDue, J. S.—(1955) *Proc. Soc. exp. Biol., N.Y.*, **90**, 210.